# What a Storehouse Is the Voluntary Hospital!

**Published:** 1914-10-10

**Authors:** 


### WHAT A STOREHOUSE IS THE VOLUNTARY HOSPITAL!

And greatly joyed merry tales to faine
Of which a storehouse did with her remain.
Spenser.

The voluntary hospital had its origin in the desire of Christian men and women to secure adequate provision for those who from sickness or accident, due to no fault of their own, required the skilled assistance of able practitioners and the security and care which an organised sick-house under proper management could alone supply to the majority of the people. Its establishment cleared the streets in some of the larger centres of population from sights which were distressing to the citizens, harmful to the health, and demoralising to the morals of the sick and injured. The voluntary hospital, therefore, became at its birth a storehouse of kindly motives expressed in good works and skilled relief, and no doubt had its origin largely in the parable of the Good Samaritan, which to-day has lost none of its influence over the hearts and consciences of the great mass of the people throughout the British Islands. We might give a most interesting account of the actual state of the early types of voluntary hospitals in this country, an idea of which, so far as their equipment and the aspects presented by their wards are concerned, is graphically exhibited in a full-page coloured illustration in R. Akerman's "Microcosm of London," vol. ii., facing page 132. Here is given a coloured illustration of a ward in the Middlesex Hospital, the first British hospital to admit lying-in women, as it was in fact 170 years ago. The interest excited by this picture is supreme for those who have studied the evolution of the modern, up-to-date voluntary hospital, especially if they have the privilege of being associated with one of these great institutions at the present time.

Our subject to-day deals directly, however, with the modern, up-to-date, voluntary hospital considered as a storehouse full of inspiration for the observant, of causes for thankfulness for the large-hearted, and of encouragement for those who believe in the inherent goodness of the British race and the influence for good of the British people wherever they may dwell. Every such institution contains the completest and most efficient apparatus for the effective use of applied science in the treatment of disease and the cure of human infirmity. There is no education so directly and quickly beneficial to an uninitiated but intelligent visitor as a careful inspection of the various departments of work which collectively constitute the establishment of a great modern hospital. The whole human family is indebted to modern scientific developments, for to them we owe, thanks largely to Lord Lister's teachings, the exhibition in our hospitals of the most perfected hygienic surroundings and the continuous enforcement of the completest cleanliness wherever disease has to be fought and health won. A mere category setting forth the names and purposes of each of the departments of a modern hospital bears eloquent testimony to the enormous developments which have taken place in the art of treatment and of the direct restoration of life, energy, and freedom from pain to an ever-increasing multitude of people through the skilled use of the surgeon's knife.

A modern hospital, therefore, when regarded from the scientific standpoint alone, exhibits abundant evidence of the justification of its description as a storehouse. The greater of our modern hospitals, which are open to the instruction and training of medical students and nurses, year by year in increasing volume and importance, become storehouses of knowledge. Previous to the present war many leaders of public thought, and those genuinely interested in the spiritual progress of the people of these islands, were full of anxiety for the future of our race, owing to the absence of depth and the lack of interest and thoroughness which characterised so large a number of the rising generation in the attainment of knowledge. The voluntary hospital from the moment it became a great centre of education—scientific, technical, moral, mechanical, and personal—has as a necessary consequence increased yearly in value as a storehouse and centre of knowledge. Who can estimate, nay, who can exaggerate, the value to the race and to humanity everywhere of the knowledge gathered up by experience and practised in a great modern hospital for the sick? All this knowledge and every addition made to it is transmitted through able teachers—some of the greatest and most proficient scientists and the most devoted and skilled members of the nursing profession—to those who, when equipped with knowledge and depth in the practice of their profession by the skill they have acquired in the hospital, go forth into the world to carry on the beneficent work amongst all who may fall out on life's journey when struck down by sickness or overtaken by accident. To-day the knowledge garnered and preserved in the hospital storehouse is systematised and reduced to order upon a carefully thought out plan which makes it readily available for use at all times, not only by members of the present generation, but for those who are to follow.

We have no space to deal with the hospital storehouse from the point of view of its lessons and influence for good, and of knowledge inestimable in value to the nation which it inculcates and spreads, through the laymen who become associated with its management, through the officers who there receive a training in the technical and difficult art of superintending and enforcing efficiency throughout its administration, and who learn here for the first time how to build up the required revenue from voluntary sources; to husband the resources thus entrusted to them, and to enforce everywhere the strictest economy combined with the most liberal and generous treatment for each patient, in accordance with his needs. These sections of the hospital storehouse have attracted, and will, we have no doubt, continue to attract, some of the busiest and ablest workers in the country.

